# Delivery, immediate newborn and cord care practices in Pemba Tanzania: a qualitative study of community, hospital staff and community level care providers for knowledge, attitudes, belief systems and practices

**DOI:** 10.1186/1471-2393-14-173

**Published:** 2014-05-22

**Authors:** Usha Dhingra, Joel Gittelsohn, Atifa Moh’d Suleiman, Shekhia Moh’d Suleiman, Arup Dutta, Said Mohammed Ali, Shilpi Gupta, Robert E Black, Sunil Sazawal

**Affiliations:** 1 Department of International Health, Johns Hopkins Bloomberg School of Public Health, Room E 5521, 615 North Wolfe Street, Baltimore, MD 21205, USA; 2 Public Health Laboratory-Ivo de Carneri, Wawi, Chake-Chake, Pemba, Zanzibar, Tanzania; 3 Center for Public Health Kinetics, New Delhi, India

**Keywords:** Newborn health, Cord care, Community, Delivery, Breastfeeding, Traditional practices

## Abstract

**Background:**

Deaths during the neonatal period account for almost two-thirds of all deaths in the first year of life and 40 percent of deaths before the age of five. Most of these deaths could be prevented through proven cost-effective interventions. Although there are some recent data from sub-Saharan Africa, but there is paucity of qualitative data from Zanzibar and cord care practices data from most of East Africa. We undertook a qualitative study in Pemba Island as a pilot to explore the attitudes, beliefs and practices of the community and health workers related to delivery, newborn and cord care with the potential to inform the main chlorhexidine (CHX) trial.

**Methods:**

80 in-depth interviews (IDI) and 11 focus group discussions (FGD) involving mothers, grandmothers, fathers, traditional birth attendants and other health service providers from the community were undertaken. All IDIs and FGDs were audio taped, transcribed and analyzed using ATLAS ti 6.2.

**Results:**

Poor transportation, cost of delivery at hospitals, overcrowding and ill treatment by hospital staff are some of the obstacles for achieving higher institutional delivery. TBAs and health professionals understand the need of using sterilized equipments to reduce risk of infection to both mothers and their babies during delivery. Despite this knowledge, use of gloves during delivery and hand washing before delivery were seldom reported. Early initiation of breastfeeding and feeding colostrum was almost universal. Hospital personnel and trained TBAs understood the importance of keeping babies warm after birth and delayed baby’s first bath. The importance of cord care was well recognized in the community. Nearly all TBAs counseled the mothers to protect the cord from dust, flies and mosquitoes or any other kind of infections by covering it with cloth. There was consensus among respondents that CHX liquid cord cleansing could be successfully implemented in the community with appropriate education and awareness.

**Conclusion:**

The willingness of community in accepting a CHX cord care practice was very high; the only requirement was that a MCH worker needs to do and demonstrate the use to the mother.

**Trial registration:**

ClinicalTrials.gov: NCT01528852

## Background

With forty-one deaths per 1000 live births, the risk of neonatal death is highest in sub-Saharan Africa. Each year, 51,000 newborns die in Tanzania, placing it among the top five countries in terms of newborn deaths in Africa [1]. Tanzania’s newborn deaths represent 29 percent of all child deaths in Tanzania. Although, Tanzania has made great strides in reducing child mortality, it has demonstrated slower progress in reducing neonatal deaths [2]. In order to meet Millennium Development Goal (MDG) 4 for child survival, newborn deaths must be reduced.

Eighty five percent of these newborn deaths in Tanzania are due to three main causes: severe infections, primarily sepsis and pneumonia (28 percent); birth asphyxia (26 percent); and complications of preterm birth (27 percent) [1]. The rest of neonatal deaths stem from factors like poor maternal health, inadequate care during pregnancy, inappropriate management of complications during delivery, and lack of newborn care. In addition, many traditional practices, such as application of substances to the stump cord, letting the baby stay wet and cold, poor hygiene during delivery and the first hours after birth, discarding colostrum and feeding other foods, can also lead to serious infections.

Many of these neonatal deaths could be averted with simple preventive measures, such as hygienic care at birth and during the postnatal period. Since the umbilical stump blood vessels are exposed for the first few days after birth, they are a common portal of entry for invasive bacteria that cause systemic infections (sepsis) in newborn babies [3], which may lead to death. In order to reduce the risk of sepsis originating from the cord stump, World Health Organization (WHO) currently recommends keeping the cord clean and dry. They additionally recommend use of topical antiseptics to the cord stump in settings where risk of infection is high. Of the numerous potential topical products (e.g., ethanol, silver sulfadiazine, triple dye, gentian violet, chlorhexidine, povidine iodine), chlorhexidine is a broad spectrum antiseptic agent that has been used extensively in hospital and other clinical settings for many decades. Recent community level randomized controlled trials in Nepal, Pakistan, and Bangladesh have shown that applying a 4% chlorhexidine product (7.1% chlorhexidine digluconate) to the umbilical cord saves lives [4-7].

All of these studies have been conducted in populations from South East Asia but evidence of cord cleansing with chlorhexidine from large randomized controlled studies from sub-Saharan Africa is lacking. In view of this we proposed to carry out a double blind controlled trial in Pemba, Zanzibar to evaluate the efficacy of application of chlorhexidine on umbilical cord of neonates during first 10 days of life. There has been paucity of data regarding community attitudes, beliefs and behaviors during delivery and newborn care from the region. We felt the need to carry out an initial formative/qualitative phase to explore the current knowledge, attitudes and practices with regard to delivery, newborn and cord care before implementation of the randomized controlled trial. This formative phase was aimed 1) To collect information on delivery practices and to understand the existing neonatal and umbilical cord care practices in the community; 2) To get feedback, perceptions and suggestions from traditional birth attendants, community members and health professionals regarding liquid cleansing solution as an umbilical cord care practice; 3) To evaluate the acceptance and barriers for the use of the proposed chlorhexidine cleansing solution; and 4) To develop communication messages, study procedures and the framework for implementing a cord care intervention based on the information gathered. The present paper is presenting the findings from this formative research. We believe that the insight into current delivery, newborn and cord care practices prevalent in Pemba, Zanzibar will help in making policy decisions which can have impact in improving newborn survival and in achieving MDG4.

## Methods

### Study setting

The study was conducted in four districts of Pemba Island, the smaller of the two islands of the Zanzibar archipelago situated 50 kilometres east of mainland Tanzania. The island has a population of about 350 000, most of whom are Afro-Shiraji Muslims, and has a tropical climate. A baseline census, including a birth history for women of reproductive age, indicated an infant mortality rate of 89 per 1000 live births [8].

### Study design

The study utilized a mixed methods approach to better understand the beliefs and practices of the community in relation to delivery, newborn care and umbilical cord care. Participants in the FGDs and IDIs were selected using a combination of purposive and random sampling. As part of qualitative research method, 80 in-depth interviews (IDIs) were conducted. In addition, 11 focus group discussions (FGDs) were held to triangulate findings from the indepth interviews. A total of 180 individuals participated in the study either as IDI participants (80) or FGD participants (100). The reason for adopting a mixed approach of using IDIs and FGDs was to ensure that triangulated responses actually represent the reality and not be affected by interviewee bias.

### Sampling and sample size

This study was based on principles of qualitative research. Final sample size was based on the principle of achieving data saturation. Data collection continued until data saturation was reached. We identified 6 significant key players contributing to maternal and newborn care (Hospital staff, PHU staff, registered TBA, unregistered TBA, mothers, grandmothers). There are 4 districts on the island and to account for variation in each of these strata by district, participants were selected from each strata from all 4 districts. A minimum of 8 respondents were selected for each strata to account for data saturation.

### Sampling strategy

**Figure 1:**
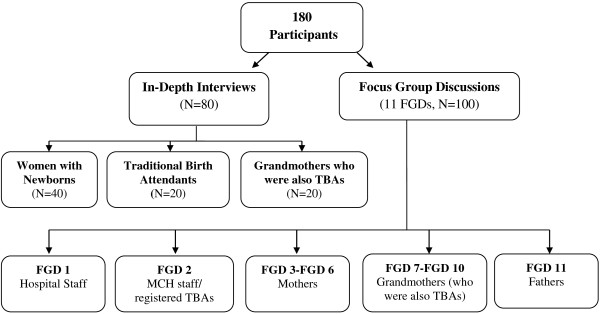
Sampling design.

To identify women of reproductive age, two villages from each district were randomly selected. From the census data available with the local institution, five women of child bearing age who had given birth to at least one child in the year preceding survey were chosen randomly from each of these villages. In all, 80 in-depth interviews were conducted including 40 women of child bearing age, 20 elderly women (grandmothers), 20 untrained and trained Traditional birth Attendants (TBAs) (Figure 1). Trained TBAs have been part of the earlier Govt. program and were provided training and delivery kits to conduct safe delivery while untrained TBAs are community based workers undertaking deliveries as part of the community service.

A total of 11 FGDs were organized, with each group having 8 participants. FGDs included hospital staff (FGD1), Maternal Child Health (MCH) staff/registered TBAs (FGD2), mothers (FGD3-FGD6), grandmothers (who were also TBAs, FGD7-FGD10) and fathers (FGD11).

### Data collection

Five health workers who had been involved in an earlier Global Burden of Disease Ethnography study were contacted and trained to do in-depth interviews and focus group discussions using the guidelines prepared by research team (Additional File 1: Focus Group Discussion and In-depth Interview Guidelines). The study instruments were pre-tested by health workers among themselves to check their appropriateness and to modify them so as to make them understandable by respondents. All the final forms and guidelines were translated into Swahili, the local language and each trained health worker conducted 2 mock interviews at Public health Laboratory (PHL) in the presence of the trainers.

### In-depth interview process

In-depth interviews consisted of open ended questions, free listing and structured questions. The IDIs were one-on-one interviews held in local language (Swahili) between a trained interviewer and study participant. A prior appointment was sought from the participant. On the day of appointment the interviewer visited the selected respondent’s household at the time preferred by the respondent. Interview lasted for 1.5 hours and was audio taped. The interviewer noted down key points of the interview in her diary. Interviewers were also asked to record interesting verbal and non verbal expressions of the participants and keep a note of local terms used.

### Focus group discussion process

Focus Group Discussions were moderated by two health workers: one acting as a Focus Group Leader (FGL) who introduced the topics of discussion in a programmed order and another one as a note keeper who was taking notes of all key points of the discussion in her diary. FGL posed the questions to the group and then asked each participant one by one if h/she would like to express her view on the topic. FGDs typically lasted for 90 minutes and were conducted in Swahili. All the focus group discussions were audio taped.

### Data analysis

Members of the field team went through the notes and audio tapes in the evening to transcribe the discussions and interviews in Swahili which were then translated into English. A thematic approach was used to analyze the transcripts [9]. It is one of a cluster of methods that focus on identifying patterned meaning across a dataset. Interviews and FGDs were read by research team and based on the texts, codes were developed and coded along thematic issues. Coded segments were examined to identify significant broad themes. New codes were developed to refine the broad themes into more specific themes. Two investigators independently applied codes to the interviews using ATLAS ti 6.2 qualitative data analysis software [10] and all final codes and themes were mutually agreed upon. The investigator team collectively selected the themes and representative quotes that were considered relevant to this publication.

### Ethical approval

The study was approved by the Johns Hopkins Institutional Review Board and the Zanzibar Research Council. Verbal Informed consent was taken from all the participants.

## Results

### Delivery practices: delivery place

Both mothers and TBAs considered hospital deliveries to be safer and better for handling of complications that might arise during delivery. Serious complications like bleeding before delivery, delayed placenta, infectious disease (like AIDS), etc. can only be handled at the hospitals. Some of the mothers were concerned about the skills of TBAs. All TBAs interviewed said that they always recommend the women to deliver at hospital but they have to deliver the women at home in case of emergency or some women insist to deliver at home.

“There are a lot of problem appeared nowadays. That’s why many women prefer to deliver at hospital. Also the TBA has not enough skills or knowledge for conducting delivery. Even little problems TBA cannot solve. For instance, the TBA might be faced with cord bleeding, and will then become frustrated as she may not know what to do.” -(FGD with mothers)

However, there were regional differences in regard to preference for birth place. In urban areas, the hospital was the place of choice while in rural areas, majority of women preferred to deliver at home either because of poverty or because of poor accessibility to health care services.

Several mothers explained that they delivered at home because of habit or tradition. Among the major reasons cited for choosing home as their preferred delivery place were: problems in transportation, past experience of safe delivery at home, lack of time to reach hospitals and excessive expenditure incurred at hospitals. Rude treatment and unavailability of beds at hospitals were cited as the prime reason for not delivering at hospitals by women.

“Some women do not prefer to go to hospital to avoid bad words from the doctors. Doctors use abusive language to their patients. Also our infrastructure is very poor. We have no road; poor transport will lead most of women to deliver at home. If you want to go to hospital, husband of the pregnant mother will hire a car which costs about 30,000/=and this is very expensive. That’s why it seems to mothers that it’s better to take delivery at home.”- (FGD with mothers)

“It is true that the delivering mothers are insulted beyond words at the hospital.”- (FGD with fathers)

### Pre-delivery preparations

Women in the community reported certain things that they keep ready with them which might be useful at the time of delivery. These include gloves, plastic sheet, plain cloth, thread and razor which they boil and store in an alcohol bottle. Coconut oil, soap and Kanga (new cloth for wrapping baby) were kept for post delivery care of newborn. They also kept some food items that they eat after delivery such as ginger for making tea, rice, sugar and flour to make porridge.

### Things to hasten delivery process

Pregnant women in the community are given specific food items, felt to aid in the delivery process. These include tea, porridge, honey, *Kombe* (a kind of holy water prepared by ‘Sheikh” i.e. Muslim leader), *Mpatakuva* (kind of herb used for abdomen treatment), traditional medicine such as roots of rose tree, henna tree and jasmine tree, bush herbs. Some other things that were used to induce labor included *Tonga,* a kind of wild fruit obtained at sea shore. Some of them also mentioned bathing and drinking cold water as strategies to hasten the delivery process. Women were advised to walk around so that the baby can easily come down into the birth passage.

“Mostly we gave them porridge and tea so that the mother can have strength, and also I tell them to walk around to make the baby delivered very easily.”-(IDI with TBA)

### Hand washing/glove wearing

There was a clear consensus for washing of hands after delivery, but the practice of washing hands before delivery and glove usage was not universally followed. Many women who had their past deliveries at home informed that majority of the TBAs used gloves for conducting delivery but expressed skepticism about the practice of hand washing before delivery.

“After finishing (the delivery), the TBA must wash her hands. She washes them by using water and soap, and sometimes should wash/bath the whole of her body and change her clothes.”- (FGD with mothers)

“I don’t know whether she washes her hands or not, but I think she only wears gloves and start to massage me.”-(IDI with mother)

These findings were confirmed during in-depth interviews with TBAs. ‘*I wear gloves, I boil thread and blade, and after delivery, I take off gloves and wash my hands and clean the mother and baby.”-(IDI with TBA)*

One of the mother informed, delay in passing the message regarding labor pain initiation to the TBA as one of the reasons for not washing hands before delivery. She described that when TBAs reach their home, the pregnant women is already in the process to deliver because of which they do not get enough time to wash their hands.

“Sometimes TBA do not wash their hands. More often TBA just wear gloves or sometimes do not wear even gloves, she just make hurry to conduct delivery. In my view, I think pregnant mother themselves delays to inform and to call the TBA. When they come the baby have already started to come out i.e the head start to come out. So, the TBA make hurry to conduct delivery and not to wear gloves.”-(FGD with mothers)

### Delivery surface

When asked about the surface of delivery, women reported giving birth on a variety of surfaces including beds with rubber sheets at the hospital, on a mattress or on the floor at home. Most women including mothers, TBAs and grandmothers reported bed as the most frequently used place for delivery at home. Some of them used to cover the bed with mackintosh just as in hospital emphasizing the need for a clean delivery surface as a means of preventing infection to their babies; however those who found it expensive simply covered the bed with old cloth.

“I tell them to lay down on the bed as what they did at Hospital.”-(IDI with TBAs)

Soon after delivery, baby was kept on bed where it was delivered until the delivery process gets completed: *“I put the baby on the bed, and I wrap her/him with a new Kanga.”-(IDI with TBA),* other women and health care providers report that the baby is immediately placed on their mother’s belly to provide him warmth from mother’s body. *“After delivery, I put the baby on the mother’s belly, then I take scissors, I tie the cord on two sides, the mother’s side, and the baby’s side, then I cut the cord.”-(IDI with TBA)*

### Rituals performed at birth

Observance of certain kind of rituals at the time of birth was quite common in the community. For instance, women who had delayed pregnancy or miscarriage in the past used to make a vow (known as *Nadhiri*) for conducting certain ceremony when they will deliver. So, they perform “Maulid Ceremony” to fulfill their vow, or distribute cooked food to the community.

“Sometimes we perform Maulid but not for every child, mostly it’s performed when the mother have complication and delivered safely.”-(IDI with mother)

One of the TBAs also reported a special ritual on the 7th day after delivery.

*“In old days, we used to make special ritual on the 7*^*th*^*day after delivery whereby we used to take the baby out. We used to put the baby in the winnowing basket, then we throw water on the roof, after that, we take the baby outside.”-(IDI with TBA)*

### Newborn care practices: bathing and cleaning of newborn

Delaying the first bath of baby soon after birth is an important practice to prevent hypothermia. In Pemba, this practice was consistently performed among hospital deliveries mainly because hospital personnel discouraged immediate bathing. For deliveries taking place at home, TBAs were primarily responsible for giving first bath to newborns, however the study revealed differences in this practice performed by the TBAs trained by the government programs and that by untrained TBAs. Untrained TBAs reported that after delivery they clean the babies and then give them bath immediately with warm water, sometimes with soap also. The trained TBAs instead of giving bath immediately after birth, only wiped the babies. Some of them also reported to practice kangaroo care. However even among them, bath was delayed by barely 6-12 hours.

“Immediately after delivery the TBA cut the cord then she wiped the baby with dry clothes and she gave her bath.”-(IDI with mother delivered by untrained TBA)

“I didn’t bath the baby at that time because we were told at the Hospital not to bath the baby immediately after delivery, they advise us we just have to wipe the baby then after some hours, the baby might be bathed.”-(IDI with trained TBAs)

Triangulation in the Focus Group Discussions further clarified that in case of hospital deliveries, although the baby was not given bath at the hospital but they bathed the baby as soon as they reached home. In case of home deliveries, the baby was bathed immediately after birth.

“If we have not stayed at the hospital for a long time. We bathe the child when we get back to home”-(FGD with mother)

Subsequently the newborn was dressed up with new clothes (*Kanga*). Following this, newborn babies were bathed regularly during the first week of birth, usually two times a day i.e. morning and evening. Warm water, soap and basin were normally used for bathing. Some of them also reported use of *Jimbo*, a mixture of various traditional herbs. However, they didn’t tell the names of those herbs as they were supposed to be kept confidential. After bath, babies were warmed using charcoal and oil massage. Majority of them used coconut oil although some of them also used olive oil and petroleum jelly. One of the TBAs also reported use of coconut oil mixed with the herbs of a tree named *Mchocha* to prevent the child from a fever called *Babu*.

“The care that was given to the baby within one week of delivery was that I used to bath him twice a day, in morning and evening. I warmed him with coconut oil and fire steam for 40 days.”-(IDI with mother)

### Breastfeeding and colostrum

In Pemba, breastfeeding is a common practice and most of the women breastfed their babies within 24 hrs of birth.

“I started to breast feed him after being cleaned and given a call for prayer “ADHANA” which was almost 2 hours after delivery.”-(IDI with mother)

TBAs and women in the community were well aware of the nutritive value of breast milk. Women in the present study widely knew about the colostrum and its health benefits. Some of the TBAs were trained by the Ministry of Health where they were taught about the nutritive value of colostrum and they encourage the mothers to feed first milk to the babies.

“On baby’s side, I tell them to just breastfeed them, they have to give nothing else to the baby even water because the breast milk is full in nutrition for the baby.”-(IDI with TBA)

Mother’s who were unable to secrete milk were generally advised to eat octopus which in their belief, helps in production of milk. Some of them did not breastfeed their babies if they suffered from HIV, had severe abdominal pain or had infection like fungus on their breast.

### Cord care practices

The umbilical cord was cut soon after delivery and mostly it was TBAs who cut the cord in case of home deliveries. The importance of a clean cord cut was commonly understood and all TBAs generally used sterile scissors or a new razor to cut the cord. Almost all TBAs informed that they disinfected these delivery instruments (razors, scissor and thread) by boiling them in water.

“After the woman delivers, I boil the blade and roll the thread, then I tie the baby’s cord by that thread and cut the cord by using the boiled blade. After cutting the cord we clean the baby and wrap in to the clean clothes”- (IDI with TBA)

TBAs further informed that razors were used only once for cutting and were buried after use.

“If we use the blades, we burry it after using, if we use the scissor we clean it by using hot water.” -(IDI with TBA)

After cutting, umbilical cord was usually tied with normal tailoring thread; few of them also reported use of special thread provided by the hospital reiterating the importance of clean cord care. Respondents described the importance of tying the cord to stop bleeding as well as to prevent infection. Some of them also reported use of Utembo, a kind of thread obtained from a palm tree called Muale.

### Cord length

There was mixed perception about the length at which cord should be cut, some of them gave no importance to cord length, according to them cord could be cut at any size while others reported that it should be cut at particular length mentioning it as an important part of delivery process.

“It doesn’t matter because everyone cut it in her own way, on my side I cut it compared with the length of my mid finger (you know one type of rice, but various type of cooking).”-(IDI with TBA)

“Yes it is important; if you cut it too short, once it is untied how you can tie it again.”-(IDI with mother)

### Cord care

The importance of cord care was well recognized in the community. Nearly all TBAs counseled the mothers to protect the cord from dust, flies and mosquitoes or any other kind of infections by covering it with cloth. The same was further confirmed by mothers during in-depth interview. Some mothers in the community informed that in case of male babies, they cover the surrounding area of the cord with cloth so as to prevent it from falling on the genital parts as they believe that if the cord falls on babies’ private parts, he will get impotent.

“They say that for a boy, to protect him, to avoid the cord to fall on his “secret parts”. They put a cloth around it. They say that if it falls on his secret part (sexual part) he may become impotent.”-(IDI with TBA)

The interviews revealed that while various newborn care programs running in the area have talked about the cord, no program mentions about the raw area left after the cord falls off. So as soon as the cord falls off, a void is formed and all sorts of traditional beliefs and practices thrust in especially if the wound is wet. In-depth interviews as well as triangulation from focus group discussions conveyed that the practice of dry cord care had percolated down to the grass root level. Following cord cutting, most of the mothers reported either using nothing or application of array of substances including: Saliva (*Mate*), dirty door powder from old door (*Ganda La Popoo*), hot knife, Charcoal powder, Shells (*Gamba La Koa*), also PPF powder, burning wood (*Kijinga*), banana steam (*Tojo*), fish bone etc. During bathing, cord was mildly cleaned, dried immediately and was covered with a cloth to protect it from flies and fiddling by older siblings.

“We don’t put anything, just apply oil and the baby powder only, we don’t put baby powder to the cord, until the cord is fallen off itself.”- (FGD with mothers)

The normal expected time for cord falling was reported to be 3-10 days. However they do not intervene if the cord takes longer to fall off but simply refer to hospital, which reflect the positive aspect of the community behavior towards the cord care. Once the cord fall-off, TBAs reported either application of door dust, charcoal powder, baby powder 444, spirit or nothing on the left out area, mainly to prevent it from infection. Cord was normally buried and some even reported to plant a coconut tree on it.

“I don’t advise them to use anything nor charcoal powder nor dirty door powder as other do, but in my area, I have educated them so they do not apply anything.”-(IDI with TBA)

### Cord care in case of infection

Development of early symptoms of cord infection is thought to be an important proximal event in the pathway to sepsis and death in newborns. The common symptoms for cord infection reported are swelling, pus formation, redness or bleeding. Mothers informed that upon recognition of any of these danger signs, they contact TBAs who usually provide home remedies, such as application of hot knife, door dust, charcoal powder, sandalwood powder, ground sea shell, PPF or talcum powder and fire steam. Some of the respondents had the perception that cord infection is caused when the mother ate certain kind of fish or other food items. In such cases, they find out the food item causing that infection, burn it and put it on the cord. Failing these home remedies or traditional care, families opt for formal medical care at a nearby public facility or by a private doctor.

“Some time the cord might be affected by “WICHO” or “KILIMI” if that happens I ask them to find something that cause that infection “WICHO”, burn it and put on the cord. It can be banana, or sea shells “KORONGONJO’. Recently there was one case happened, the infant’s cord was having infection, the baby was taken to hospital but the baby’s cord didn’t cure, I told them to burn the stump of banana and put on the baby’s cord and it recovered”- (IDI with TBAs)

### Perceptions and suggestions about implementation of liquid cord cleaning

There was a clear consensus among the health professionals, TBAs and community members in regard to the successful implementation of liquid cord cleaning. There was an emphasis on creating awareness in the community about usefulness of CHX in cord cleaning; educating the community before implementing CHX cord care. While some of the mothers and TBAs had a view that CHX should be applied by health professionals, others were confident that if mothers are properly trained they can apply the CHX on baby’s cord themselves.

“If people are educated on the use of this antiseptic solution they will be willing to use it. – (IDI with mother)

“My side I think it should be used and mother can apply it herself”- (IDI with TBAs)

“Liquid based cleansing solution is a good idea, but the people should be educated and there is no problem for mothers to apply it if they get instructions”- (FGD with mothers)

“This thing is new to us. I myself have never heard about this medicine. In the past they used to apply dust from the door on the cord. Now that is out of date. Mothers use modern medicines. Hence if there is such a medicine and researches have been made to see that it is safe to use it we ask the Ministry to allow us to use it and we will be willing to use it” – (FGD with MCH)

### Barriers/constraints to liquid cord cleaning

As part of the FGDs and IDIs respondents were specifically asked about the possible constraints in implementation of CHX as liquid cord care. Almost all the respondents were of the opinion that there will not be any constraints while very few respondents cited few concerns. Lack of understanding, cost of CHX solution and religious beliefs were cited as some of the potential issues in the community.

“No constraints” - (IDI with mother)

“There will not be any problem” - (FGD with TBAs)

“Some people may have constraints in introducing this cord care practice on ground that they may think it is against their custom” – (IDI with mother)

### Findings from structured component of interviews

As part of the structured questionnaire relating to cord care, application of liquid antiseptic solution to cord, the perceptions of mothers were; 88% of the women did not know about the use of antiseptic solution for cord care and yet 99% were willing to use the liquid cord care solution. 93% of respondents were of the opinion that mothers can apply the CHX solution themselves insisting that they should be trained by MCH workers. The only constraints mentioned were cost of CHX if people have to buy it themselves (11.3%), lack of understanding (7%) and religious customs (2.8%). 69% of the respondents felt that there will not be any impediment to CHX liquid cord care.

(The detailed data pertaining to structured interview component are presented as Additional file 2: Supplementary Tables).

## Discussion

Appropriate antenatal, intra-partum and post-natal care play a crucial role in preventing mortality and in achieving optimal health outcomes for infants and young children. The current study is one of the very few qualitative studies on selected delivery and newborn care practices conducted in East Africa (The only one conducted in Zanzibar). The study in addition to focusing on issues around delivery, newborn care practices complementing the data from other recent studies in Sub Saharan Africa [11,12] and Tanzania mainland [13] added a new dimension of cord care practices and perceptions regarding use of CHX as antiseptic cord care. Zanzibar has been under Omani influence for more than two centuries and therefore perceptions and practices might be different between Tanzania main land and Zanzibar.

The level of perinatal mortality rate (PNMR) in a community is said to be associated with the proportion of births that take place at a health facility and the coverage of skilled attendance at birth. Countries where skilled attendance and institutional delivery rates are low usually have a high PNMR [14]. Hospital deliveries accounted for majority (64.3%) of the births in this study. TBAs in the community were generally aware of the problems of delivering at home which can be easily handled at hospitals. Similar findings have been reported in another study conducted in Pemba [15].

The finding of the present study suggests that although there is improvement in the proportion of those delivering at hospitals in Pemba, they should be further encouraged to deliver at hospitals in efforts to reduce child mortality. Poor transportation facilities were found as one of the major hurdles in this direction which forced many families in the community to hire cars to reach hospitals which is generally very expensive. Bang *et al*. [16] have proven the feasibility of a low-cost approach of delivering primary neonatal care at home using intensive support by locally trained community health workers in India. Similar approach can be replicated in other poor resource countries like sub-Saharan Africa. Another hurdle observed in the present study was cost of delivery at hospitals. Increasing healthcare utilization among these communities would require eliminating this unnecessary cost, further, health care providers should be trained in proper patients dealing so as to make hospitals ‘mother-child friendly’. Lack of knowledge about antenatal care is another hurdle in newborn and maternal health as finding of the study suggests that majority of the mothers who had not gone for antenatal visit were actually not aware about it.

Studies from rural areas have underscored the role of family members during delivery and care of newborn [17,18]. Such a practice highlights the importance of providing training to family members regarding delivery process which would better equip them in dealing with delivery complications.

According to the World Health Organization, the risk of umbilical cord infection increases when unclean materials are used to cut the cord [3]. In the present study, almost all TBAs reported using a sterilized blade, which is different than other studies conducted in rural area of Bangladesh [19] and Nepal [17]. Similarly, use of sterilized thread to tie the cord was also very common in the community. They were generally aware that unsterilized equipment was a major risk factor for cord infection. Thus, the public health recommendations to use sterilized equipment for delivery purpose fits well with the local cultural understanding.

The time immediately following delivery is a vulnerable period for both the woman and the baby. WHO has recommended thermal control of newborn in the essential newborn care [20]. In the present study, the practice of bathing newborns immediately after birth or simply wiping them off with a warm towel varied according to the place of delivery and training of the TBAs. TBAs who were trained regarding neonatal care generally avoided bath immediately after birth as compared to untrained TBAs. Bathing was also delayed when babies were born at hospitals. However, even in case of trained TBAs, delay was not more than 24 hrs as more than 70% of the newborns were given bath within 24 hrs after delivery. Further, there is a practice of regular bathing of newborns during the first few days after delivery, usually twice daily, which increases the risk of hypothermia. This practice is similar to those in other countries in the South Asian region [17,18].

On the positive side, mothers and TBAs in Pemba were concerned about the protection of newborns from cold and took great measures to keep them warm in the days following delivery. Immediately after birth, newborns are wrapped in new clean cloth (*Kanga*) and given bath with warm water and occasionally with some traditional herbs (*Jimbo*) to protect the baby from fever and cold. After bath, baby was kept warm with charcoal and coconut oil. Some traditional herbs were also added to the oil for protection from fever. However, use of traditional treatments such as exposing the newborns to charcoal fumes for warming should be avoided as exposing newborns to smoke could put them at risk of respiratory problems.

The present study showed that Pemba is one of the places where an optimal dry cord care is practiced. There are very few deviations which include application of dirty door powder from old door (*Ganda La Popoo*), hot knife, Charcoal powder, Shells (*Gamba La Koa*), also PPF powder, burning wood (*Kijinga*), banana steam (*Tojo*), fish bone etc. The main lacuna seems to be after falling of the cord where the health messages are not clear. Women reported a range of substances that were applied to umbilical stump. However, application of such harmful therapies in the management of babies exacerbates the probability for the development of infections like tetanus, omphalitis, fever, septicemia, etc. Earlier studies from Nepal and Pakistan also reported use of similar materials on the newborn’s umbilical cord highlighting the prevalence of this practice at a wider geographical range [17,21,22].

Exclusive breastfeeding is an important behavior that should be initiated within the first hour after birth and maintained until the newborn is six months old. In the present study, breastfeeding practices seem to be adequate and healthy. The rate of initiation and exclusive breastfeeding seems to be healthy and encourageous, which is higher than the percentages reported in other studies [23]. We did not discover any negative perceptions about feeding colostrum except for one TBA. This finding has positive implications for infant nutrition. The only instance when mothers disagree to breastfeed was when they have some problem like severe abdominal pain, infection etc. Thus, the finding that breast-milk is highly nutritious and should be given immediately after birth, is clearly in accordance with the current global health recommendations.

### What we learnt from the study to inform the main CHX application trial

After analyzing the data collected from this phase we realized that although most of the community was unaware of CHX antiseptic solution they were positive about its use as a cord care solution emphasizing that community should be educated about its usefulness. Community members believed that first CHX application should be done by hospital staff/MCH worker rather than the community health worker. MCH workers should demonstrate/instruct mothers as to the application of CHX solution on the cord and then the mothers can do it themselves. This was an important finding which lead us to re strategize the implementation of the main trial. We had initially planned to use community workers for application of CHX for the main trial but based on these findings engaged the MCH workers for CHX application on day 0, 1, 4 and 10. They taught the mothers on CHX application and mothers applied it on the rest of the days.

### Limitation of the study

The current study was based on reported practices and not on actual observation and hence was subject to recall and response bias. Hence, the interpretation and generalizability of our findings may be limited. It is possible that some of the health functionaries reported what they were expected to practice in comparison to what they actually did.

### Implications of the study

Despite the above-mentioned limitations, our study has obtained important information about delivery and newborn care practices. This information will assist in planning public health interventions to change these behaviors. Some of the practices reported in this study benefit the mother and the newborns and hence should be encouraged. This includes use of clean delivery instruments, TBAs’ emotional and physical support to mothers, newborn warming and immediate breastfeeding. Unfortunately, some behaviors inadvertently undermine newborn survival like jeopardizing mother from antenatal care, immediate bathing of newborn risking hypothermia and removal of protective vernix, cleanliness of TBAs (unclean hands), exposing the newborn to smoke for warming, application of some traditional substances on cord etc. and therefore should be discouraged.

Our study suggests that sheer availability of maternity services may not be enough to ensure the use of such services. Lack of utilization may be influenced by income, poor infrastructure in the form of transportation, hospital staff’s behavior and traditional beliefs. These issues need to be addressed in order to promote hospital based deliveries which would in turn assist in reducing newborn mortality.

## Conclusion

In Pemba the awareness among the community regarding importance of institutional deliveries seems to be very high. However impediments as regards to transportation and care at the facilities seemed to be a reason for this positive knowledge not resulting in appropriate practice with women choosing to deliver at home. The willingness of community in accepting a CHX cord care practice was very high; the only requirement was that a MCH worker needs to do and demonstrate the use to the mother. The current study highlighted very subtle and important issues that would help improve success of currently ongoing programs and also helped modifications in the design of main CHX efficacy trial which was hence successfully implemented with completion due in next 6 months.

## Abbreviations

CHX: Chlorhexidine; IDI: In-depth interviews; FGD: Focus group discussions; WHO: World health organization; MGD: Millennium development goal; TBA: Traditional birth attendants; MCH: Maternal child health; PHL: Public health laboratory; FGL: Focus group leader; PHU: Primary health unit; AIDS: Acquired immunodeficiency syndrome; HIV: Human immunodeficiency virus; PNMR: Perinatal mortality rate.

## Competing interests

Authors declared no competing interest.

## Authors’ contributions

UD was involved in the development of the study questionnaires, guidelines, training of interviewers, coding and analysis of data, and the preparation of manuscript. JG helped with the development of study questionnaires, guidelines, analysis and preparation of manuscript. AMS was involved in primary data collection as well as the transcription and translation of data. SMS was involved in primary data collection as well as the transcription and translation of data. AD helped with the development of study questionnaires, guidelines and field management. SMA helped with the Pemba administrative system, community mobilization and provided leadership and supervision to team members. SG helped with the analysis and manuscript writing. REB was involved in the conceptualization of the research and the preparation of the manuscript. SS was involved in the conceptualization of research, development of study instrument, development of study protocol, coding and analysis of data, and the preparation of manuscript. All authors read and approved the final manuscript.

## Pre-publication history

The pre-publication history for this paper can be accessed here:

http://www.biomedcentral.com/1471-2393/14/173/prepub

## Supplementary Material

Additional file 1 Focus Group Discussion and In-depth Interview Guidelines. Click here for file

Additional file 2 Supplementary Tables. Click here for file

## References

[B1] Lawn J Kerber K Opportunities For Africa’s Newborns: Practical Data, Policy And Programmatic Support For Newborn Care In Africa 2006 Cape Town: PMNCH, Save the Children, UNFPA, UNICEF, USAID, WHO

[B2] Masanja H de Savigny D Smithson P Schellenberg J John T Mbuya C Upunda G Boerma T Victora Smith T Mshinda H Child survival gains in Tanzania: analysis of data from demographic and health surveys Lancet 2008 371 1276 1283 10.1016/S0140-6736(08)60562-0 18406862

[B3] WHO Care Of The Umbilical Cord: A Review The Evidence 1998 Chapter 1 1 (WHO/RHTMS/98.4)

[B4] Mullany LC Darmstadt GL Khatry SK Katz J LeClerg SC Shrestha S Adhikari R Tielsch JM Topical applications of chlorhexidine to the umbilical cord prevent omphalitis and neonatal mortality in southern Nepal: a community-based, cluster randomized trial Lancet 2006 367 910 918 10.1016/S0140-6736(06)68381-5 16546539 PMC2367116

[B5] Mullany LC Darmstadt GL Tielsch JM Role of antimicrobial applications to the umbilical cord in neonates to prevent bacterial colonization and infection: a review of the evidence Pediatr Infect Dis J 2003 22 996 1002 10.1097/01.inf.0000095429.97172.48 14614373 PMC1317298

[B6] Soofi S Cousens S Imdad A Bhutto N Ali N Bhutta ZA Topical application of Chlorhexidine to neonatal umbilical cords for prevention of omphalitis and neonatal mortality in a rural district of Pakistan: a community-based, cluster-randomised trial Lancet 2012 379 1029 1036 10.1016/S0140-6736(11)61877-1 22322126

[B7] Goldenberg RL McClure EM Saleem S A review of studies with chlorhexidine applied directly to the umbilical cord Am J Perinatol 2012 30 699 701 23254380 10.1055/s-0032-1329695 PMC3875170

[B8] Sazawal S Black RE Ramsan M Chwaya HM Stoltzfus RJ Dutta A Dhingra U Kabole I Deb S Othman MK Kabole FM Effects of routine prophylactic supplementation with iron and folic acid on admission to hospital and mortality in preschool children in a high malaria transmission setting: community-based, randomised, placebo-controlled trial Lancet 2006 367 133 143 10.1016/S0140-6736(06)67962-2 16413877

[B9] Braun V Clarke V Using thematic analysis in psychology Qual Res Psychol 2006 3 2 77 101 10.1191/1478088706qp063oa

[B10] ATLAS.ti Scientific Software Development. Atlas Ti Version 6 2011 Berlin, Germany: ATLAS.ti Scientific Software Development GmbH

[B11] Moyer CA Aborigo RA Logonia G Affah G Rominski S Adongo PB Williams J Hodgson A Engmann C Clean delivery practices in rural northern Ghana: a qualitative study of community and provider knowledge, attitudes, and beliefs BMC Pregnancy Childbirth 2012 12 50 10.1186/1471-2393-12-50 22703032 PMC3482570

[B12] Hill Z Manu A Tawiah-Agyemang C Gyan T Turner K Weobong B Ten Asbroek AH Kirkwood BR How did formative research inform the development of a home-based neonatal care intervention in rural Ghana? J Perinatol 2008 28 Suppl 2 S38 S45 19057567 10.1038/jp.2008.172

[B13] Mosha F Winani S Wood S Changalucha J Ngasalla B Evaluation of the effectiveness of a clean delivery kit intervention in preventing cord infection and puerperal sepsis among neonates and their mothers in rural Mwanza Region, Tanzania Tanzan Health Res Bull 2005 7 3 185 188 16941946 10.4314/thrb.v7i3.14258

[B14] Lawn JE Cousens S Zupan J 4 million neonatal deaths: When? Where? Why? Lancet 2005 365 891 900 doi: 10.1016/S0140-6736(05)71048-5 pmid: 15752534 10.1016/S0140-6736(05)71048-5 15752534

[B15] Thairu L Pelto G Newborn care practices in Pemba Island (Tanzania) and their implications for newborn health and survival Matern Child Nutr 2008 4 194 208 10.1111/j.1740-8709.2008.00135.x 18582353 PMC6860798

[B16] Bang AT Bang A Baitule SB Reddy MH Deshmukh MD Effect of home-based neonatal care and management of sepsis on neonatal mortality: field trial in India Lancet 1999 354 1955 1961 10.1016/S0140-6736(99)03046-9 10622298

[B17] Osrin D Tumbahangphe KM Shrestha D Mesko N Shrestha BP Manandhar K Standing H Manandhar DS Costello AM Cross sectional, community based study of care of newborn infants in Nepal BMJ 2002 325 1063 1066 10.1136/bmj.325.7372.1063 12424164 PMC131178

[B18] Thapa N Chongsuvivatwong V Geater AF Ulstein M High-risk childbirth practices in remote Nepal and their determinants Women Health 2000 31 83 97 11310813 10.1300/j013v31n04_06

[B19] Darmstadt GL Syed U Patel Z Kabir N Review of domiciliary newborn care practices in Bangladesh J Health Pop Nutr 2006 24 380 393 17591335 PMC3001142

[B20] WHO Thermal Control Of The Newborn: A Practical Guide 1993 Geneva: Maternal Health and Safe Motherhood Programme, Division of Family Health, WHO

[B21] Moran AC Choudhury N U Zaman Khan N Ahsan Karar Z Wahed T Rashid SF Alam AM Newborn care practices among slum dwellers in Dhaka, Bangladesh: a quantitative and qualitative exploratory study BMC Pregnancy Childbirth 2009 9 54 10.1186/1471-2393-9-54 19919700 PMC2784437

[B22] Fikree FF Ali TS Durocher JM Rahbar MH Newborn care practices in low socioeconomic settlements of Karachi, Pakistan Soc Sci Med 2005 60 911 921 10.1016/j.socscimed.2004.06.034 15589663

[B23] Masvie H The role of Tamang mothers-in-law in promoting breast feeding in Makawanpur District, Nepal Midwifery 2006 22 23 31 10.1016/j.midw.2005.02.003 15967547

