# Supplementary material for: Delivery, immediate newborn and cord care practices in Pemba Tanzania: a qualitative study of community, hospital staff and community level care providers for knowledge, attitudes, belief systems and practices
Source: BMC Pregnancy Childbirth. 2014 May 22;14:173. doi: 10.1186/1471-2393-14-173 (PMC4049503; doi:10.1186/1471-2393-14-173)
Supplement: Additional file 1 — Focus Group Discussion and In-depth Interview Guidelines. [file 1471-2393-14-173-S1.doc]

FOCUS GROUP DISCUSSION GUIDE FOR

MCH STAFF/SKILLED TBAS

**Purpose:** To explore current common practices, beliefs and attitude regarding maternal and newborn care among MCH staff and Skilled Birth Attendants and triangulate information collected as part of one to one interviews.

**Methods:** Focus group discussion

**Materials:** Pen & paper, MP 3 recorder

**Date**_________________

**District** _________________ **Location** _________________

**Time: from**_________________ **to**_________________

**Facilitator** ____________________________________

**Note taker** _____________________________________

**Number of participants** _______________________

### Preparation for Focus Group

- Focus groups would consist of 16 people
  - - - Focus groups would be diverse.
      - Participants would be selected to represent different socioeconomic and demographic background.
- Translate survey instrument and probe questions into local language
  - Protocols for translation, back-translation
    1. Have two translators translate the entire instrument separately from English to local language.
    2. Have two different translators translate the entire instrument back separately from the local language to English without seeing the original English version.
    3. Have the four translators work together to address any discrepancy in the translation and produce a final translated instrument.
- Preparation of Venue
- Arrange the room so that it is conducive to discussion (e.g. chairs in a circle) and privacy is maintained (so that the conversation will not be overheard) and there will be no interruptions.
- Prepare and test audio recording equipment

**Instructions for the Facilitator:**

1. Greet participants, make them feel comfortable. Thank them for taking time to join the discussion.

[As the moderator, you need to feel comfortable and guide the discussion in a very natural way. You need to remember that some people are by nature more talkative and opinionated than others. As the moderator, you need to make sure the silent ones get a chance to express themselves, and that the more talkative do not dominate the discussion. At the same time, remember that a FGD is a dynamic process that depends on the interaction among participants and the discussion among them. Try to discourage all participants from talking at the same time, but do not discourage the discussion among them.]

2. Introduce yourself and the note taker.

3. Now I would like each of you to introduce yourself to the group.

4. Explain to the participants why you have called them to this discussion. Read the following statement.

[“I have asked you to join us to discuss current maternal, neonatal and cord care practices in order to improve the health of the new born children in Pemba. I am particularly interested to know the beliefs and practices in the community associated with delivery and neonatal care. We will introduce you to the use of an antiseptic solution to clean the umbilical cord of the newborn and would like to get your opinion on the feasibility of using it in the community. Please feel free to talk. Your opinions are very important and there is no right or wrong answer. We will use your responses to develop the communication package for use among women during ANC, delivery and postnatal period. Remember, this is just a discussion and every opinion counts. We are interested in everyone’s ideas and hope that you will give each other the opportunity to participate.” As you discuss we will be taking notes of everything you say. Your answers will be kept confidential and only the researchers and study personnel will have access to this information. The interview will take between 1 to 1.5 hours and will be conducted so as to guarantee your privacy. May I proceed with the discussion? Yes/No. For those agreeing to participate, sign in the consent forms along with the participant’s name and date.

5. Tape recorder. “Because the note taker will not be able to write down all that you say, I need to rely on a tape recorder. I will listen to this tape this evening and write down what you said. (**Place emphasis on the importance of tape recording and reassurance of total confidentiality to increase the participant’s level of cooperation in this regard).**

6. Focus Group Discussion Questions: Your role as moderator/discussion leader is to make sure all questions are covered, and to keep the flow of the discussion within the scope of the subject matter. Probe questions are asked only if the group discussion does not address the issues of the probe].

**Objectives**

1. To understand the beliefs and perceptions of MCH/Skilled TBAs regarding delivery, newborn and cord care practices.
2. To understand the delivery, post partum and newborn care experiences of mothers in the community.
3. To understand willingness and concerns that the community may have in the use of a liquid cleaning solution for cord care.

Q1. What is your view on the use of contraception? In Pemba in last few years has it increased in the community or is it the same?

Q2. Do you think that some women want to use contraception and are not able to do it ? What are the reasons for that?

Q3. Do you have contraception supplies and If yes, where and how are they distributed in the community?

Q4. What is the practice of antenatal care in the community? Where do most of the pregnant women go for antenatal check-ups? What facilities are available at MCH clinics? How many visits are planned and how many visits pregnant women make?

Q5.What proportion of women delivers at home and has it changed in last few years?

Q6. In the interviews we have realized that most women would like to deliver at hospital? Is that true? What is the reason for that?

Q7. In which situation delivery takes place at home?

Q8. At what time is the decision made regarding who will deliver the woman and where?

Q9. At what time is the TBA/MCH staff informed about pregnancy?

Q10. In our interviews we have learnt that women who have problems inform TBA and MCH earlier. Can you tell us under which circumstances they contact TBA/ MCH staff earlier?

Q11. Do you have pregnancy confirmation test available with you? How many women contact you for confirmation of pregnancy and what do you do in that case?

Q12. We realized from the interviews that soap is not listed among things kept ready for delivery? Is it because soap is already available at home or is not important?

Q13. How often the person conducting delivery wash hands and with what?

Q14. We have realized that these days TBAs/MCH take mothers to hospital for deliveries. How often does this happen? Why? What happens after reaching the hospital?

Q15. If there are complications and mother is unconscious or is very sick what happens to the baby? Who takes care of the baby and where?

Q16. What happens if a child is born premature? Do you take special care of these babies and what advice do you give?

Q17. We have found in the interviews that nearly always the blade and the thread are boiled before delivery. Does person conducting delivery always boil these things before delivery or there are times when it is not done?

Q18. What are the terms that you use If a child is born dead? Are there different terms if a child after birth has movement/breathes/ or cry and then dies?

Q19. If the child is born at hospital how long do mothers stay at the hospital after normal delivery? How long does TBA/MCH staff who took the mother to hospital stay at the hospital?

Q20. What are the factors that determine the length of stay at the hospital after delivery?

Q21. When a TBA conducts delivery at home how long does she stay at home after delivery? What does she do during that time?

Q22. For hospital delivery after the baby is born what all is done to the child in next one hour after birth?

Q23. Can you describe when a bath is given at birth or in first one hour what all is done like what all is used to bath, what is used to dry the baby, use of soap etc.

Q24. At the time of cleaning/bathing baby in first hour then what do you do to cord? (Do you wash the cord, Do you clean the cord-how?, apply something to the cord)

Q25. What all do you observe in the baby to make sure that the baby is alright during first hour after birth?

Q26. What is done to the baby on the first day after birth in terms of cleaning/bathing/applying substances/ massages?

Q27. What is done to the cord on day after delivery in terms of cleaning/bathing/wrapping and things applied?

Q28. After delivery how many visits do you make the mother and the baby and when?

Now let us talk about time between day 2 and till the time cord fall off

Q29. What all is done to the baby in terms of bathing/cleaning/massage/things applied

Q30. What all is done to the cord in terms of cleaning/bathing/ wrapping/ things applied?

Q31. In the time between delivery and till the time cord fall off -If you observe that baby has swelling/ redness / pus / foul smelling discharge or bleeding from the cord stump what do you do and what are the things that you apply as remedies?

[Introduce cord cleaning with liquid solution]

Recently you participated in the pre testing of the fluid application of cord our feedback was that it was successful without problems but we would like to get you opinion. Can you give us your suggestion as to how we can make it better for main trial? As you know that we tested three different containers- Which one you think was better and why?

Q32. Finally can you help us understand what would be the best system to identify all pregnancies and possibly to identify them early. e.g. Do we need survey / should we have education campaigns so that pregnancies get reported to MCH/TBA

FOCUS GROUP DISCUSSION GUIDE FOR MOTHERS/GRANDMOTHERS/UNSKILLED TRADITIONAL BIRTH ATTENDANTS

**Purpose:** To explore current common practices, beliefs and attitude regarding maternal and newborn care among the mothers.

**Methods:** Focus group discussion

**Materials:** Pen & paper, MP 3 recorder

**Date**_________________

**District** _________________ **Location** _________________

**Time: from**_________________ **to**_________________

**Facilitator** ____________________________________

**Note taker** _____________________________________

**Number of participants** _______________________

### Preparation for Focus Group

- Focus groups would consist of 16 people
  - - - Focus groups would be diverse.
      - Participants would be selected to represent different socioeconomic and demographic background.
- Translate survey instrument and probe questions into local language
  - Protocols for translation, back-translation
    1. Have two translators translate the entire instrument separately from English to local language.
    2. Have two different translators translate the entire instrument back separately from the local language to English without seeing the original English version.
    3. Have the four translators work together to address any discrepancy in the translation and produce a final translated instrument.
- Preparation of Venue
- Arrange the room so that it is conducive to discussion (e.g. chairs in a circle) and privacy is maintained (so that the conversation will not be overheard) and there will be no interruptions.
- Prepare and test audio recording equipment

**Instructions for the Facilitator:**

1. Greet participants, make them feel comfortable. Thank them for taking time to join the discussion.

[As the moderator, you need to feel comfortable and guide the discussion in a very natural way. You need to remember that some people are by nature more talkative and opinionated than others. As the moderator, you need to make sure the silent ones get a chance to express themselves, and that the more talkative do not dominate the discussion. At the same time, remember that a FGD is a dynamic process that depends on the interaction among participants and the discussion among them. Try to discourage all participants from talking at the same time, but do not discourage the discussion among them.]

2. Introduce yourself and the note taker.

3. Now I would like each of you to introduce yourself to the group.

4. Explain to the participants why you have called them to this discussion. Read the following statement.

[“I have asked you to join us to discuss current maternal, neonatal and cord care practices in order to improve the health of the new born children in Pemba. I am particularly interested to know the beliefs and practices in the community associated with delivery and neonatal care. We will introduce you to the use of an antiseptic solution to clean the umbilical cord of the newborn and would like to get your opinion on the feasibility of using it in the community. Please feel free to talk. Your opinions are very important and there is no right or wrong answer. We will use your responses to develop the communication package for use among women during ANC, delivery and postnatal period. Remember, this is just a discussion and every opinion counts. We are interested in everyone’s ideas and hope that you will give each other the opportunity to participate.” As you discuss we will be taking notes of everything you say. Your answers will be kept confidential and only the researchers and study personnel will have access to this information. The interview will take between 1 to 1.5 hours and will be conducted so as to guarantee your privacy. May I proceed with the discussion? Yes/No. For those agreeing to participate, sign in the consent forms along with the participant’s name and date.

5. Tape recorder. “Because the note taker will not be able to write down all that you say, I need to rely on a tape recorder. I will listen to this tape this evening and write down what you said. (**Place emphasis on the importance of tape recording and reassurance of total confidentiality to increase the participant’s level of cooperation in this regard).**

6. Focus Group Discussion Questions: Your role as moderator/discussion leader is to make sure all questions are covered, and to keep the flow of the discussion within the scope of the subject matter. Probe questions are asked only if the group discussion does not address the issues of the probe].

**Objectives**

1. To understand the beliefs and perceptions of mothers regarding delivery, newborn and cord care practices.
2. To understand the delivery, post partum and newborn care experiences of mothers in the community.
3. To understand willingness and concerns that mothers/grandmothers in the community may have in the use of a liquid cleaning solution for cord care.

Q1. What is your view on the use of contraception? In Pemba in last few years has it increased in the community or is it the same?

Q2. Do you think that some women want to use contraception and are not able to do it ? What are the reasons for that?

Q3 What is the practice of antenatal care in the community? Where do most of the pregnant women go for antenatal check-ups? What facilities are available at MCH clinics?

Q4.What proportion of women delivers at home and has it changed in last few years?

Q4. In the interviews we have realized that most women would like to deliver at hospital? Is that true? What is the reason for that?

Q5. In which situation delivery takes place at home?

Q6. At what time is the decision made regarding who will deliver the woman and why?

Q7. At what time is the TBA informed?

Q8. In our interviews we have learnt that women who have problems inform TBA and MCH earlier. Can you tell us under which circumstances they contact TBA earlier?

Q9. In the interviews we have come to know of things that women collect for delivery- what we would like to know is that do they buy them separately or is there a package available containing all the things together? How much is the cost?

Q10. We realized from the interviews that soap is not listed among things kept ready for delivery? Is it because soap is already available at home or is not important?

Q11. How often the person conducting delivery wash hands ?

Q12. We have realized that these days TBAs take mothers to hospital for deliveries. How often does this happen? Why? What happens after reaching the hospital?

Q13. If there are problems during delivery either at hospital or at home what happens to the baby? Who takes care of the baby and where?

Q14. What happens if a child is born premature? Do you take special care of these babies?

Q15. We have found in the interviews that nearly always the blade and the thread are boiled before delivery. Does person conducting delivery always boil these things before delivery or there are times when it is not done?

Q16. What are the terms that you use If a child is born dead? Are there different terms if a child after birth has movement/breathes/ or cry and then dies?

Q17. If the child is born at hospital how long do mothers stay at the hospital after normal delivery?

Q18. What are the factors that determine the length of stay at the hospital after delivery?

Q19. When a TBA conducts delivery at home how long does she stay at home after delivery? What does she do during that time?

Q20. For hospital delivery after the baby is born what all is done to the child in next one hour after birth?

Q21. Can you describe when a bath is given at birth or in first one hour what all is done like what all is used to bath, what is used to dry the baby, use of soap etc.

Q22. At the time of cleaning/bathing baby in first hour then what do you do to cord? (Do you wash the cord, Do you clean the cord-how?, apply something to the cord)

Q23. What all you observe in the baby to make sure that the baby is alright during first hour after birth?

Q24. What is done to the baby on the first day after birth in terms of cleaning/bathing/applying substances/ massages

Q25. What is done to the cord on day after delivery in terms of cleaning/bathing/wrapping and things applied

Q26. Now let us talk about time between day 2 and till the time cord fall off

Q27. What all is done to the baby in terms of bathing/cleaning/massage/things applied ?

Q28. What all is done to the cord in terms of cleaning/bathing/ wrapping/ things applied?

Q29. In the time between delivery and till the time cord fall off -If you observe that baby has swelling/ redness / pus / foul smelling discharge or bleeding from the cord stump what do you do and what are the things that you apply as remedies?

Q30. We have been told in the interviews that when the cord falls down on pubic area it causes sterility in males. Is it true for females also or it does not matter in case of females?

[Introduce cord cleaning with liquid solution]

There is some evidence from Asia that application and cleaning of cord with an antiseptic solution may have beneficial effect and reduce illness and deaths in newborns. In a project which will start soon, this beneficial effect is proposed to be tested in Pemba as well. I would like us to discuss your views in regard to use of this antiseptic solution

Q31. What is your view on the implementation of this program? Can mother/caretaker apply it herself?

IN DEPTH INTERVIEW DISCUSSION GUIDE FOR MOTHERS/GRANDMOTHERS

**Purpose:** To explore current common practices, beliefs and attitude regarding maternal and newborn care among the women of childbearing age who had given birth to a child previously.

**Methods:** One to One interview

**Materials:** Pen & paper, MP 3 recorder

**Date**_________________

**District** _________________ **Village** _________________

**Time: from**_________________ **to**_________________

**Instructions for the Interviewer:**

1. Visit the scheduled household, greet the woman and read the following script

[“I want to thank you for taking out time to meet me today. My name is _____________ and I work at the PHL-IdC. I would like to know and record your views about delivery, postnatal, and newborn care in the community. I will introduce you to the use of an antiseptic solution to clean the cord and would like to get your opinion on the feasibility of using it in the community. The interview will take about 1.5 hours. I will be taping the session because I don’t want to miss any of your comments. Although I will be taking some notes during the session, I can’t possibly write fast enough to get it all down. Because we’re on tape, please be sure to speak up so that we don’t miss your comments. All responses will be kept confidential. This means that your interview responses will only be shared with research team members and we will ensure that any information we include in our report does not identify you as the respondent. Remember, you don’t have to talk about anything you don’t want to and you may end the interview at any time.

Are there any questions about what I have just explained?

Are you willing to participate in this interview?

2. Interview Questions: Please make sure that all questions are covered, and keep the flow of the discussion within the scope of the subject matter. Ask Probe questions only if the woman does not address the issues of the probe.

[Let us start with your experience in regard to delivery, new born and cord care and perceptions and practices in the community]

**Part A: Open ended questions**

Q1. Please tell me how many members are there in your family?

How many children do you have?

Can you tell me the name of your youngest child?

How old is your youngest child?

. (ice breaker intended to generate rapport)

Please tell me about what steps/measures you took before delivery of (name of youngest child). (Probe on when is the first contact made with TBA, how information about her getting into labor was passed, who conducted the delivery, where was it conducted? in case of hospital delivery what things did she take to the hospital, in case of home delivery what things were kept ready?)

Tell me about what are the things that happened during the delivery of (name of youngest child).

Q2. How was the decision regarding where delivery should take place made, who made it and what factors influenced that decision? (Probe planned site of delivery, emergency selection)

Q3. Can you tell us when a child is born what all is done to him or her immediately upon birth, during first few hours and then during first 24 hours after birth.

(Probe on what is done to the infant on the birthday in regard to cleaning, bathing, and religious ceremonies)

Q4. Now that we have passed and understood the first day, can you describe what do you do to take care of baby during first week in terms of cleaning, feeding, religious ceremonies, safety and wellbeing?

Q5. Can you now tell me what care you received immediately after delivery and in the week following delivery for your well being? (Probe in terms of medical checkup, feeding, rest, bathing, cleanliness, diet, visitations, and religious)

Q6. Were any of your children born in the hospital? If yes, can you describe to me your experience during the hospital delivery?

Q7. Tell me how soon you started breastfeeding your (youngest child) and for how long did you breastfeed?

Q9. Please tell me the problems that may occur at the time of delivery and how they are dealt with in this community? (Probe Prolonged labor, emergency transport to health facility)

Q10. What are the symptoms, which are considered to indicate that baby, is not well after delivery and what is done if these occur? (Probe signs, local names and responses to each)

Q11. Let us now talk about the stump of the cord left on the abdomen of the baby after cord is cut. What are the local terms used to refer to it and what is done to take care of it after delivery, in next 24 hours and in next 7-10 days? (Probe on length/cleaning/ wrapping/ apply something)

Q12. What is done after cord stump has fallen off (Probe on if there is a time expectation within which it should fall off and if it does not what do people do?).

**Part B: Free List:**

**(These will be appropriately translated into local language)**

1. What are all the different illnesses of newborn children that you can think of? (once you have the list, probe on causes and/or symptoms for each, which ones can cause death, what can be done to prevent these?)
2. What kind of traditions and customs are observed at and immediately after birth?
3. What things are used to massage the mother after birth?
4. What things are used to massage the baby after birth?
5. What is used to bathe the baby? (Free List)
6. What are all the different things that are put on the cord of new born that you can think of? (Free list)
7. What kind of topical substances are applied on the skin of new born?
8. What are the local terms used for umbilical cord infections?
9. What are commonly used traditional remedies for cord infections?

**Part C: Semi Structured Survey Questionnaire**

1. In your community, where do most of the women deliver?

- Home
- Hospital
- PHCU or MCH Clinic
- Other place ______________________(specify)

1. Do family members decide about place of birth in advance?
   - Yes
   - No
2. In the community, who conducts the delivery? (check all that apply)

- Skilled TBA
- Unskilled TBA
- Relative
- Neighbor
- Elderly women
- Others

1. When do families make first contact with TBA?

- When woman gets into labor
- Early in Pregnancy
- Late in Pregnancy

1. When woman gets into labor, who informs the TBA/Woman who conducts delivery?

- Family members
- Relatives
- Neighbors
- Others

1. What do the TBAs/ delivery woman do for cleanliness before delivery (tick all that applies)?

- Wear gloves
- Wash hands
- Use soap
- Dry hands

1. What is used for cutting the cord of the baby?
   - Regular shaving blade
   - Knife
   - Sterilized blade
   - Scissor
   - Others ____________________
2. What is used to tie the cord after it is being cut (Discuss options)

- Thread
- Clamp
- String from a piece of cloth
- Others _______________________

1. What kind of surface is used for delivery? ____________________________
2. In case of hospital deliveries, who generally accompanies the woman to the hospital?

- Mother in law
- Husband
- Neighbor
- Elderly woman
- Relative
- Friend
- Mother
- TBA
- Other

1. What kind of transportation is used to go to hospital?

- Dala Dala
- Scooter
- Car
- Bullock carts
- Other _________________________________

1. Is delivery kit normally used for delivery?

- Does not know about delivery kit
- Why/Why not ___________________________________________

**Immediate and post delivery care**

[Now I would like to discuss with you about the care given to your (youngest) baby immediately after birth and within first month]

1. Where was (name of the youngest baby) placed after birth?

- Floor as it is
- Floor washed and cleaned
- Bed
- Others---------------------------

1. When was (name of the youngest baby) cleaned after birth?

- Immediately
- After some time when cord has been cut
- Others………………………..

1. With what was the (name of the youngest baby) cleaned after birth?

- Water
- Water and soap
- Moist cloth
- Wiped with cloth only
- Jimbo
- Others …………………

1. Who cleaned the baby?

- Grand mother
- TBA
- Relative
- Mother
- Hospital attendant/nurse
- Other

1. When was the first bath given to the baby?

- immediately
- within in 24 hours
- after 24 hours

1. Who gave the bath?

- TBA
- Hospital attendant/nurse
- Mother/Mother-in-law
- Grand mother
- Relative
- Neighbor
- Other

1. Was Warm/cold water used?
   1. Warm
   2. Cold
2. Was something added to the water?
   1. Yes
   2. No

If yes what…………………..

1. Was something applied to the eyes?
   1. Yes
   2. No
   3. If yes what………………….
2. What was used to dry and wrap the baby?

- Towel
- Old cloth
- Kanga

[Let us talk about breastfeeding and other feeding practices]

1. How long after birth was the baby breastfed?

- immediately
- Within 24 hours
- after 24 hours
- Not breastfed

1. Did you feed the baby with colostrums?

- Yes
- No
- Milk did not come

1. How long after birth did you get to see the (name of the youngest baby)?

- Immediately
- After _______ hours/days

1. Were you advised to take special diet after giving birth to (name of the youngest baby)?
   1. Yes
   2. No
   3. If yes what……………
2. Were you given massage?

- For how many days? ________
- What material was used for massage ………….

1. Was (name of the youngest baby) given massage?

- Yes
  1. For how many days? ________
  2. What material was used for massage ______________
- No

1. For how many days were you advised to take rest? ______
2. Did someone come to examine you and the baby after birth?
   1. Yes, immediately after birth
   2. Yes, few hours after birth
   3. No
3. How many days after birth were you allowed to go out of home?

_______ (Days)

1. Did you perform any religious function for the new baby?
   1. Yes, How many days after birth? _______
   2. No

21. What was fed to (name of the youngest baby) during first month of life?

- Breast milk?
- Water?
- Tea?
- Honey?
- Chew dates?
- Glucose
- Light Porridge
- Cow milk
- Formula Milk
- Others

**Umbilical Cord care**

[Now let us talk about umbilical cord care.].

1. Does the length of cord matter?

- Yes ___________________________________________________
- No

1. Were the instruments used for cutting the cord cleaned before use?

- Yes
- No

1. If yes to Q2, how were these instruments cleaned?

- Boiled
- Burnt in fire
- already sterilized

1. Were the materials used for tying the cord available at home?

- Yes
- No

1. Was something applied on the cord after the cord was cut?

- Yes
- No
- If yes, what_____________________

1. If answer to Q5 is Yes, ask who applied these substances

- Mother
  - - Grand mother
    - Relative
    - Neighbor
    - TBA
    - Other

1. If answer to Q5 is Yes, why were these substance applied?
   - to help cord heal early
   - for health
   - to stop bleeding
   - Others
2. What was done to the cord after it had fallen off?

- burnt
- buried
- kept safely at home
- others ______________________________

1. How many days did it take for (name of the youngest child) cord to fall off? __________
2. Do you think it matters if cord takes longer than usual to fall off?

- Yes
- No

1. Did you apply anything after the cord had fallen off?

- Yes
- No
- If yes, what…………..

1. If answer to Q11 is Yes, how long did you apply? _________
2. Did you observe any rituals with the falling of the cord?

- Yes
- No
- Describe………………

1. What do you do if baby’s cord has redness or swelling or pus formation?

- Apply home made remedies
- take advice from relative/neighbor
- take to hospital/health facility
- talk to TBA

1. Do you think it as a serious cause of illness

- Yes
- No

**Part 2. Introduction of a liquid based cleansing solution for umbilical cord care**

**Interviewer:**

Introduce cleaning of umbilical cord and stump with Chlorhexidine Solution

[There is some evidence from Asia that application and cleaning of cord with an antiseptic solution may have beneficial effect and reduce illness and deaths in newborns. In a project which will start soon, this beneficial effect is proposed to be tested in Pemba as well. I would like us to get your views in regard to use of this antiseptic solution].

1. Do you know what is an antiseptic?
   - Yes
   - No
2. Do people in the community ever use antiseptic or liquid solution to clean the cord?
   - Yes
   - No
3. If yes, when
   - In case of infection
   - Pus formation
   - In general
   - Other
4. What is your opinion about cleaning the cord with a liquid solution?

- Good, should be used to clean the cord
- No, it is bad to use liquid solution to clean the cord
- Should only be used in case of infection
- Don’t know

1. What do you think about touching the cord with hand?
   - Shouldn’t be touched at all with hand
   - Can be touched after washing the hand
   - Doesn’t matter
2. How often do you / family members check the cord?

- Everyday
- After every 2-3 days
- Doesn’t pay any special attention
- Don’t know

1. In case you have another baby and we advise you to clean the cord of the baby with a liquid solution will you be willing to do it?

- Yes
- No , Why ________________________________________________

1. Do you think family members can apply this solution or do you think there is a need for trained person?

- Yes family members can do it
- No, there is a need for a trained person

1. Do you wash hands before touching the cord?

- Yes
- No

1. What do you think is needed to successfully introduce the liquid cord cleaning as cord care practice in the community?

________________________________________________________________________________________________________________________________________________________________________________________________________________________

1. What do you think are the constraints or hurdles in introducing this cord care practice?

________________________________________________________________________________________________________________________________________________________________________________________________________________________

IN DEPTH INTERVIEW DISCUSSION GUIDE FOR TRADITIONAL BIRTH ATTENDANTS (TBAs)

**Purpose: :** To explore current common practices, beliefs and attitude regarding maternal and newborn care among the Traditional Birth Attendants.

**Methods:** One to One interview

**Materials:** Pen & paper, MP 3 recorder

**Date**_________________

**District** _________________ **Village** _________________

**Time: from**_________________ **to**_________________

**Instructions for the Interviewer:**

1. Visit the scheduled household, greet the woman and read the following script

[“I want to thank you for taking out time to meet me today. My name is _____________ and I work at the PHL-IdC. I would like to know and record your views about delivery, postnatal, and newborn care in the community. I will introduce you to the use of an antiseptic solution to clean the cord and would like to get your opinion on the feasibility of using it in the community. The interview will take about 1.5 hours. I will be taping the session because I don’t want to miss any of your comments. Although I will be taking some notes during the session, I can’t possibly write fast enough to get it all down. Because we’re on tape, please be sure to speak up so that we don’t miss your comments. All responses will be kept confidential. This means that your interview responses will only be shared with research team members and we will ensure that any information we include in our report does not identify you as the respondent. Remember, you don’t have to talk about anything you don’t want to and you may end the interview at any time.

Are there any questions about what I have just explained?

Are you willing to participate in this interview?

2. Interview Questions: Please make sure that all questions are covered, and keep the flow of the discussion within the scope of the subject matter. Ask Probe questions only if the woman does not address the issues of the probe.

[Let us start with your experience in regard to delivery, new born and cord care and perceptions and practices in the community]

**Part A: Open ended questions**

Q1. Please tell me your name

[TBA name] How did you become a TBA?

Have you been trained to be a TBA? If yes, from whom?

. (ice breaker intended to generate rapport)

Do you keep track of pregnancies in your community? How?

How many deliveries do you conduct in a week?

Are the numbers of deliveries increasing/decreasing in your community?

Please tell me how you are informed about pregnancy and what steps/measures you take before delivery of pregnant women in your community. (Probe on when is the first contact made with TBA, how many contacts does she make with the pregnant woman before delivery, what advice does she give to the pregnant woman during these contacts, how information about pregnant woman getting into labor is passed, how does she reach the pregnant woman, what advice does she give to the woman in labor, what does she give to the woman to make delivery easier)

Describe to me the steps that take place from the time you are called to attend delivery till you leave household after delivery (Probe on what preparations does she make- wash hands, wear gloves, which are the things that she keep ready, what are the things that she tells family members to keep ready, what all she tells the woman at the time of labor, if asks relatives to help).

Q2. How is the place of delivery (home or hospital) decided? Why? Can you explain to me all the possibilities?

Q3. What kind of problems you have faced during deliveries of women (Please ask for local terms for these problems)

Q4. What do you do to solve these problems (Probe on what does she do at the time of problem, note down the name of traditional medicines if she uses them, when does she advice the family to take the woman to hospital)

Q5. What are the danger signs/complications at the time of delivery (Please ask for local terms) and what do you when you encounter these problems?

Q6. What are the things you do to the woman immediately after baby has come out? (Probe on in terms of cleaning, bathing, massaging,)

Q7. Can you tell me what do you do to the baby immediately after he/she is born? (Probe in terms of where she places the baby after birth, cleaning/bathing/wrapping – how much time after birth do you clean/bathe the baby, what do you use to clean the baby, bathe the baby, wrap the baby)

Q8. What advice do you give to the family members for feeding of the new mother and baby?

Q9. What advice do you give to women about breastfeeding (Probe on when does she tell new mother to start breastfeeding- how much time after delivery, when she discourages new mother to breastfeed-why?)

Q10. What do you tell the new mothers to do with Colostrum?

Q11. How long do you stay at the time of delivery and what follow up visits you make? What do you do on the follow up visits?

Q12. What are the symptoms that indicate to you that baby is not well after delivery and what do you do to handle it? (Probe signs, local names and responses to each)

Q13. What do you do to the cord after birth? What advice do you give the mother/family member to do with the cord for the days till it fall off? What are the local terms for the cord and different conditions that may affect it like swelling, infection etc and the names used for these conditions? (Probe on how she removes the placenta, what does she do if there is problem with removing placenta, is length of cord important-why, What advice does she give to the mother for cord care)

Q14. What are the symptoms/signs that tell you that baby’s cord is infected (Note down all signs, what do you do if baby has these signs, when do you tell the family to take the baby to the hospital)

Q15. What is done after cord stump has fallen off (Probe on if there is a time expectation within which it should fall off and if it does not what do people do? Does she advice the family to apply something on the skin after cord has fallen off-What, Why).

**Part B: Free List:**

**(These will be appropriately translated into local language)**

1. Please list all the different illnesses of newborn children that you know of? (once you have the list, probe on causes and/or symptoms for each, which ones can cause death, what can be done to prevent these?)
2. Please enumerate different traditions and customs observed at and immediately after birth in the community?
3. Give me a list of things that are used to help women deliver easily? (Please note down in sequence)
4. Please give me a list of things that are used to massage the mother after birth?
5. What things are used to massage the baby after birth?
6. What is used to bathe the baby? (Please make a list)
7. Please tell me list of things that are used to cut the cord?
8. What things are used to tie the cord?
9. How do you clean the instruments used for cutting the cord?
10. What are all the different things that are put on the cord of new born (Free list)
11. What kind of topical substances applied on the skin of new born?
12. Please give me a list of local terms used for umbilical cord infections?
13. What are commonly used traditional remedies for cord infections? (Please note down in sequence)

**Part C. Introduction of a liquid based cleansing solution for umbilical cord care**

**Interviewer:**

Introduce cleaning of umbilical cord and stump with Chlorhexidine Solution

[There is some evidence from Asia that application and cleaning of cord with an antiseptic solution may have beneficial effect and reduce illness and deaths in newborns. In a project which will start soon, this beneficial effect is proposed to be tested in Pemba as well. I would like us to get your views in regard to use of this antiseptic solution].

1. Do you know what is an antiseptic?
   - Yes
   - No
   - If yes, ask her to describe and give examples ___________________________________
2. Do you ever use antiseptic or liquid solution to clean the cord?
   - Yes
   - No
3. If yes, when
   - In case of infection
   - Pus formation
   - In general
   - Other __________________________________________________________
4. What is your opinion about cleaning the cord with a liquid solution?

- Good, should be used to clean the cord
- No, it is bad to use liquid solution to clean the cord
- Should only be used in case of infection
- Don’t know

1. What do you think about touching the cord with hand?
   - Shouldn’t be touched at all with hand
   - Can be touched after washing the hand
   - Doesn’t matter
2. Do you wash hands before touching the cord?

- Yes
- No

1. Do you check the cord of the baby after birth?

- Yes
- No
- If yes, what do you check _____________________________________

1. In case we advise you to clean the cord of the baby you deliver with a liquid solution will you be willing to do it?

- Yes
- No , Why ________________________________________________

1. Do you think if you give advice to the family members to apply this solution, they will be willing to use this liquid cleaning solution

- Yes
- No Why __________________________________________________

1. What do you think is needed to successfully introduce the liquid cord cleaning as cord care practice in the community?

________________________________________________________________________________________________________________________________________________________________________________________________________________________

1. What do you think are the constraints or hurdles in introducing this cord care practice?

________________________________________________________________________________________________________________________________________________________________________________________________________________________
