# Supplementary material for: Delivery, immediate newborn and cord care practices in Pemba Tanzania: a qualitative study of community, hospital staff and community level care providers for knowledge, attitudes, belief systems and practices
Source: BMC Pregnancy Childbirth. 2014 May 22;14:173. doi: 10.1186/1471-2393-14-173 (PMC4049503; doi:10.1186/1471-2393-14-173)
Supplement: Additional file 2 — Supplementary Tables. [file 1471-2393-14-173-S2.docx]

| **Additional file 2: Supplementary Tables** | | | | |  |
| --- | --- | --- | --- | --- | --- |
| **Supplementary Table 1: Practices regarding Delivery** | | | | |  |
|  | | | | | N=80 |
| Where do most of the women deliver? | | | | | |
| Home | 35.7 | | | | |
| Hospital | 64.3 | | | | |
| Do family members decide about place of birth in advance? | | | | | |
| Yes | | 52.9 | | | |
| No | | 47.1 | | | |
| In the community, who conducts the delivery? | | | | | |
| Skilled TBA | | | 64.3 | | |
| Unskilled TBA | | | 6.7 | | |
| Elderly women | | | 2.9 | | |
| Others | | | 26.1 | | |
| When do families make first contact with TBA? | | | | | |
| When woman gets into labor | | | | 64.8 | |
| Early in Pregnancy | | | | 5.6 | |
| Late in Pregnancy | | | | 29.6 | |

| **Supplementary Table 2a : Practices related to** **Immediate newborn care** | | |  | | |  |
| --- | --- | --- | --- | --- | --- | --- |
|  | | | N=80 | | |  |
| Where is baby placed after birth? | | |  |  |  |  |
| Floor as it is | | | 1.6 | | | |
| Floor washed and cleaned | | | 3.2 | | | |
| Bed | | | 85.2 | | | |
| Other | | | 10.0 | | | |
| What is used for cutting the cord of the baby? | | |  |  |  |  |
| Regular shaving blade | | | 7.1 | | | |
| Knife | | | 5.7 | | | |
| Sterilized blade | | | 80.0 | | | |
| Scissor | | | 7.2 | | | |
| When is baby cleaned after birth? | |  |  |  |  |  |
| Immediately | 47.1 | | | | | |
| After some time when cord has been cut | | | | | 35.7 | |
| Others | 17.2 | | | | | |
| With what is the baby cleaned after birth? | |  |  |  |  |  |
| Water and soap | | | | 24.3 | | |
| Wiped with cloth only | | | | 41.4 | | |
| Others | | | | 34.3 | | |
| What is used to dry and wrap the baby | | | |  | | |
| Towel | | | | 16.1 | | |
| Old Cloth | | | | 3.2 | | |
| Kanga | | | | 80.7 | | |

| **Supplementary Table 2b: Practices regarding Immediate newborn care:** **Baby Bath and breast feeding** |  | |  |
| --- | --- | --- | --- |
|  | N=80 | |  |
| When is the first bath given to the baby? |  |  |  |
| Immediately | 20.0 | | |
| Within in 24 hours | 51.4 | | |
| After 24 hours | 28.6 | | |
| How long after birth is the baby breastfed? |  |  |  |
| Immediately | 14.3 | |  |
| Within 24 hours | 72.9 | |  |
| After 24 hours | 12.8 | |  |
| Do you feed the baby with colostrums? |  |  |  |
| Yes | 71.4 |  |  |
| No | 28.6 |  |  |

| **Supplementary Table 3a. Practices regarding Cord Handling after birth** | | | | | | | | | | | | |  |
| --- | --- | --- | --- | --- | --- | --- | --- | --- | --- | --- | --- | --- | --- |
|  | | | | | N=80 | | | | | | | |  |
| Are the instruments used for cutting the cord cleaned before use? | | | | | | | | | | | | |  |
| Yes | | | | | | | | 84.2 | | | | |  |
| No | | | | | | | | 15.8 | | | | |  |
| Is something applied on the cord after the cord is cut? | | | | | | | | | | | | |  |
| Yes | | | | | | | 17.1 | | | | | |  |
| No | | | | | | | 82.9 | | | | | |  |
| Do you think it matters if cord takes longer than usual to fall off? | | | | | | | | | | | | |  |
| Yes | | | | | | | | 61.4 | | | | |  |
| No | | | | | | | | 38.6 | | | | |  |
| Do you apply anything after the cord has fallen off? | | | | | | | |  |  |  |  |  |  |
| Yes | | | | | | | 51.4 | | | |  |  |  |
| No | | | | | | | 48.6 | | | |  |  |  |
|  | | | | | | | |  | | | | |  |
|  | | | | |  | | | | | | | | |
|  | **Supplementary Table 3 b: Practices regarding dealing with Cord Infections** | | | | | | | |  |  |  |  |  |
|  | | | | N=80 | | | | |  |  |  |  |  |
| What do you do if baby’s cord has redness or swelling or pus formation? | | | |  | | | | |  |  |  |  |  |
| Apply home remedies | | | | 8.6 | | | | |  |  |  |  |  |
| Take to hospital/health facility | | | | 88.6 | | | | |  |  |  |  |  |
| Talk to TBA | | | | 2.9 | | | |  |  |  |  |  |  |
| Do you think it as a serious cause of illness | | | |  |  |  |  |  |  |  |  |  |  |
| Yes | | | | 92.9 | | | | |  |  |  |  |  |
| No | | | | 7.1 | | | | |  |  |  |  |  |

| \| **Supplementary Table 4 a. Perceptions regarding introduction of an antiseptic liquid cord cleaning** \| \| \| \| \| \| \| \| \| \| \| \| \| --- \| --- \| --- \| --- \| --- \| --- \| --- \| --- \| --- \| --- \| --- \| --- \| \| Do you know what an antiseptic is? \| \| \| \| \| \| \| \| \| \| \| \| \| Yes \| \| \| \| 12.3 \| \| \| \| \| \| \| \| No \| \| \| \| 87.7 \| \| \| \| \| \| \| \| Do people in the community ever use antiseptic or liquid solution to clean the cord? \| \| \| \| \| \| \| \| \| \| \| \| \| Yes \| \| \| 12.0 \| \| \| \| \| \| \| \| No \| \| \| 88.0 \| \| \| \| \| \| \| \| What is your opinion about cleaning the cord with a liquid solution? \| \| \| \| \| \| \| \| \| \| \| \| \| Good, should be used to clean the cord \| \| \| \| \| \| 83.8 \| \| \| \| \| Should only be used in case of infection \| \| \| \| \| \| 8.1 \| \| \| \| \| Don’t know \| \| \| \| \| \| 8.1 \| \| \| \| \| What do you think about touching the cord with hand? \| \| \| \| \| \| \| \| \| \| \| \| \| Shouldn’t be touched at all with hand \| \| \| \| \| \| 42.5 \| \| \| \| Can be touched after washing the hand \| \| \| \| \| \| 49.3 \| \| \| \| Doesn’t matter \| \| \| \| \| \| 8.2 \| \| \| \| How often do you / family members check the cord? \| \| \| \| \| \| \| \| \| Everyday \| \| \| \| \| 95.2 \| \| \| \| \| After every 2-3 days \| \| \| \| \| 1.6 \| \| \| \| \| Doesn’t pay any special attention \| \| \| \| \| 1.6 \| \| \| \| \| Don’t know \| \| \| \| \| 1.6 \| \| \| \| \| In case you have another baby and we advise you to clean the cord of the baby with a liquid solution will you be willing to do it? \| \| \| \| \| \| \| \| \| Yes \| \| \| \| \| \| \| 98.6 \| \| \| No \| \| \| \| \| \| \| 1.4 \| \| \|  \| \| \| \| \| \| \| \| \|  \|  \| \| \| \| \| \| \| \|  \|  \| \| \| \| \| \| \| \|  \| \| \| \| \| \| \| \| \|  \|  \| \| \| \| \| \| \| \|  \|  \| \| \| \| \| \| \| \|  \| \| \| \| \| \| \| \| \|  \| \|  \| \| \| \| \| \| \|  \| \|  \| \| \| \| \| \| \|  \| \|  \| \| \| \| \| \| \|  \| \|  \| \| \| \| \| \| \|  \| \|  \| \| \| \| \| \| \| **Supplementary Table 4b. Perceptions regarding introduction of chlorhexidine cord care study** \| \| \| \| \| \| \| \| \| Do you think family members can apply this solution or do you think there is a need for trained person? \| \| \| \| \| \| \| \| \| Yes family members can do it \| \| \| \| \| 93.2 \| \| \| \| \| No, there is a need for a trained person \| \| \| \| \| 6.8 \| \| \| \| \| Do you wash hands before touching the cord? \| \| \| \| \| \| \| \| \| Yes \| \| \| \| 72.4 \| \| \| \| \| No \| \| \| \| 27.6 \| \| \| \| \| How to introduce liquid cord cleaning successfully in the community \| \| \| \| \| \| \| \| \| Should be given at hospital/clinic \| \| \| \| 40.0 \| \| \| \| \| \| People should be educated/trained/given advice \| \| \| \| 32.3 \| \| \| \| \| \| Should be with TBAs \| \| \| \| 15.3 \| \| \| \| \| \| Should be free \| \| \| \| 8.0 \| \| \| \| \| \| Others \| \| \| \| 4.4 \| \| \| \| \| \| What could be the constraints to liquid cord cleaning \| \|  \| \| \| \| \| \| \| \| No constraints \| \| \| 69.0 \| \| \| \| \| \| Could be a problem if people do not understand \| \| \| 7.0 \| \| \| \| \| \| How to get the medicine \| \| \| 1.5 \| \| \| \| \| \| Contrary to customs \| \| \| 2.8 \| \| \| \| \| \| If is sold as people cannot afford it \| \| \| 11.3 \| \| \| \| \| \| others \| \| \| 8.4 \| \| \| \| \| | |
| --- | --- | --- | --- | --- | --- | --- | --- | --- | --- | --- | --- | --- | --- | --- | --- | --- | --- | --- | --- | --- | --- | --- | --- | --- | --- | --- | --- | --- | --- | --- | --- | --- | --- | --- | --- | --- | --- | --- | --- | --- | --- | --- | --- | --- | --- | --- | --- | --- | --- | --- | --- | --- | --- | --- | --- | --- | --- | --- | --- | --- | --- | --- | --- | --- | --- | --- | --- | --- | --- | --- | --- | --- | --- | --- | --- | --- | --- | --- | --- | --- | --- | --- | --- | --- | --- | --- | --- | --- | --- | --- | --- | --- | --- | --- | --- | --- | --- | --- | --- | --- | --- | --- | --- | --- | --- | --- | --- | --- | --- | --- | --- | --- | --- | --- | --- | --- | --- | --- | --- | --- | --- | --- | --- | --- | --- | --- | --- | --- | --- | --- | --- | --- | --- | --- | --- | --- | --- | --- | --- | --- | --- | --- | --- | --- | --- | --- | --- | --- | --- | --- | --- | --- | --- | --- | --- | --- | --- | --- | --- | --- | --- | --- | --- | --- | --- | --- | --- | --- | --- | --- | --- | --- | --- | --- | --- | --- | --- | --- | --- | --- | --- | --- | --- | --- | --- | --- | --- | --- | --- | --- | --- | --- | --- | --- | --- | --- | --- | --- | --- | --- | --- | --- | --- | --- | --- | --- | --- | --- | --- | --- | --- | --- | --- | --- | --- | --- | --- | --- | --- | --- | --- | --- | --- | --- | --- | --- | --- | --- | --- | --- | --- | --- | --- | --- | --- | --- | --- | --- | --- | --- | --- | --- | --- | --- | --- | --- | --- | --- | --- | --- | --- | --- | --- | --- | --- | --- | --- | --- | --- | --- | --- | --- | --- | --- | --- | --- | --- | --- | --- | --- | --- | --- | --- | --- | --- | --- | --- | --- | --- | --- | --- | --- | --- | --- | --- | --- | --- | --- | --- | --- | --- | --- | --- | --- | --- | --- | --- | --- | --- | --- | --- | --- | --- | --- | --- | --- | --- | --- | --- | --- | --- | --- | --- | --- | --- | --- | --- | --- | --- | --- | --- | --- | --- | --- | --- | --- | --- | --- | --- | --- | --- | --- | --- | --- | --- | --- | --- | --- | --- | --- | --- | --- | --- | --- | --- | --- | --- | --- | --- | --- | --- | --- | --- | --- | --- | --- | --- | --- | --- | --- | --- | --- | --- | --- | --- | --- | --- | --- | --- | --- | --- | --- | --- | --- | --- | --- | --- | --- | --- | --- | --- | --- | --- | --- | --- | --- | --- | --- | --- | --- | --- | --- | --- | --- | --- | --- | --- | --- | --- | --- | --- | --- | --- | --- | --- | --- | --- | --- | --- | --- | --- | --- | --- | --- | --- | --- | --- | --- | --- | --- | --- | --- | --- | --- | --- | --- | --- | --- | --- | --- | --- | --- | --- | --- | --- | --- | --- | --- | --- | --- | --- | --- | --- | --- | --- | --- | --- | --- | --- | --- | --- | --- | --- | --- | --- | --- | --- | --- | --- | --- | --- | --- | --- | --- | --- | --- | --- | --- | --- | --- | --- | --- | --- | --- | --- | --- | --- | --- | --- | --- | --- | --- | --- | --- | --- | --- | --- | --- | --- | --- | --- | --- | --- | --- |
|  |  |
|  |  |
|  |  |
